# Detection of Wandering Behaviors Using a Body-Worn Inertial Sensor in Patients With Cognitive Impairment: A Feasibility Study

**DOI:** 10.3389/fneur.2021.529661

**Published:** 2021-03-11

**Authors:** Rebecca J. Kamil, Dara Bakar, Matthew Ehrenburg, Eric X. Wei, Alexandra Pletnikova, Grace Xiao, Esther S. Oh, Martina Mancini, Yuri Agrawal

**Affiliations:** ^1^Department of Otolaryngology-Head and Neck Surgery, Johns Hopkins University, Baltimore, MD, United States; ^2^Warren Alpert Medical School of Brown University, Providence, RI, United States; ^3^Johns Hopkins University School of Medicine, Baltimore, MD, United States; ^4^Division of Geriatric Medicine and Gerontology, Department of Medicine, Johns Hopkins University, Baltimore, MD, United States; ^5^Department of Neurology, Oregon Health and Science University, School of Medicine, Portland, OR, United States

**Keywords:** dementia, wandering behavior, turning, cognitive impairment, body-worn inertial sensor

## Abstract

Patients with Alzheimer's disease (AD) and AD related dementias (ADRD) often experience spatial disorientation that can lead to wandering behavior, characterized by aimless or purposeless movement. Wandering behavior has been associated with falls, caregiver burden, and nursing home placement. Despite the substantial clinical consequences of wandering, there is currently no standardized approach to objectively quantify wandering behavior. In this pilot feasibility study, we used a lightweight inertial sensor to examine mobility characteristics of a small group of 12 older adults with ADRD and mild cognitive impairment in their homes. Specifically, we evaluated their compliance with wearing a sensor for a minimum of 4 days. We also examined the ability of the sensor to measure turning frequency and direction changes, given that frequent turns and direction changes during walking have been observed in patients who wander. We found that all patients were able to wear the sensor yielding quantitative turn data including number of turns over time, mean turn duration, mean peak turn speed, and mean turn angle. We found that wanderers make more frequent, quicker turns compared to non-wanderers, which is consistent with pacing or lapping behavior. This study provides preliminary evidence that continuous monitoring in patients with dementia is feasible using a wearable sensor. More studies are needed to explore if objective measures of turning behaviors collected using inertial sensors can be used to identify wandering behavior.

## Introduction

People with Alzheimer's disease (AD) and AD-related dementias (ADRD) can experience impaired spatial awareness and navigation ability, which is thought to lead to wandering behavior (1–4). Wandering may involve repetitive movements including pacing, defined as back-and-forth movement in a limited area, and lapping, defined as repetitive walking in circuitous paths (5, 6). Wandering can also include random movements and increased duration of walking with frequent episodes of getting lost (2, 6, 7). Wandering has been associated with a myriad of negative outcomes including falls and subsequent injuries, increased caregiver burden, and early institutionalization (8–10). However, currently, there is no standardized approach to objectively describe and measure this behavior. The lack of a precise, objective metric has led to difficulty in studying the risk factors for wandering, the natural history and progression of this behavior, and effectiveness of interventions. Wandering behavior is typically detected by caregiver report, which may be imprecise, as it is based on the caregiver's ability to recognize and report this behavior. Various technologies, such as video surveillance, fluorescent dye–based image processing, wearable global positioning systems (GPSs), and electronic tagging, have been used to physically track wandering patients to help prevent elopement (5, 6, 11–14), but there is currently no standardized approach to objectively measure and quantify the wandering behavior itself.

In the past 10–15 years, wearable sensor technology in the form of inertial measurement units (IMUs) has provided a new avenue for detecting and monitoring the “quantity” and “quality” of mobility and physical activity under natural conditions in a variety of neurological diseases including dementia. These studies used IMUs that were worn by participants to record mobility patterns and quantify gait and turning through accelerations and angular velocity signals (15–20). Although these papers characterized specific impairments in quality of gait over multiple days in people with mild AD (18, 19) and reported reduced quantity of physical activity in people with dementia (21, 22), they did not report information on wandering behaviors. A recent study found that the turning behaviors in older adults with or without cognitive impairment could be successfully characterized with wearable sensors through 7 days of continuous monitoring (23). Characteristics of the turning, including number of turns per hour and speed of turning, were related to the individual's spatial cognitive abilities and also differentiated fallers from non-fallers. Since wandering behavior is associated with repetitive pacing and lapping, which likely affect the frequency and speed of turns, studying turning behavior may offer an objective way to describe the wandering behavior seen in older adults with cognitive impairment. To our knowledge, a link between turning characteristics and wandering has not been studied before. We hypothesize that quantification of turning characteristics using body-worn inertial sensors can provide an objective metric of wandering behavior. As a first step in this line of research, we aimed to assess the feasibility of using objective characteristics of turning quality in real-life conditions as a measure of wandering behavior.

## Materials and Methods

Participants were recruited from the Johns Hopkins Memory and Alzheimer's Treatment Center (JHMATC). Eligibility criteria included: (1) minimum age of 55 years, (2) a diagnosis of mild cognitive impairment (MCI) or AD based on the 2011 National Institute on Aging—Alzheimer's Association (NIA-AA) criteria (24) or other types of dementia, (3) presence of a caregiver who spends a minimum of 10 h weekly with the participant, and (4) residence within 60 miles of JHMATC given that the accelerometers were set up in patient homes. All of the study participants were diagnosed by dementia specialists at the Johns Hopkins University School of Medicine. We compared our patient data to historical control data obtained as part of a separate study by one of the study investigators (MM), where the same methodology was applied. The control participants were enrolled in the Oregon Center for Aging and Technology (ORCATECH) study of healthy aging and were free of neurological disease or dementia, in contrast to the patients enrolled in the current study.

**Figure 1:**
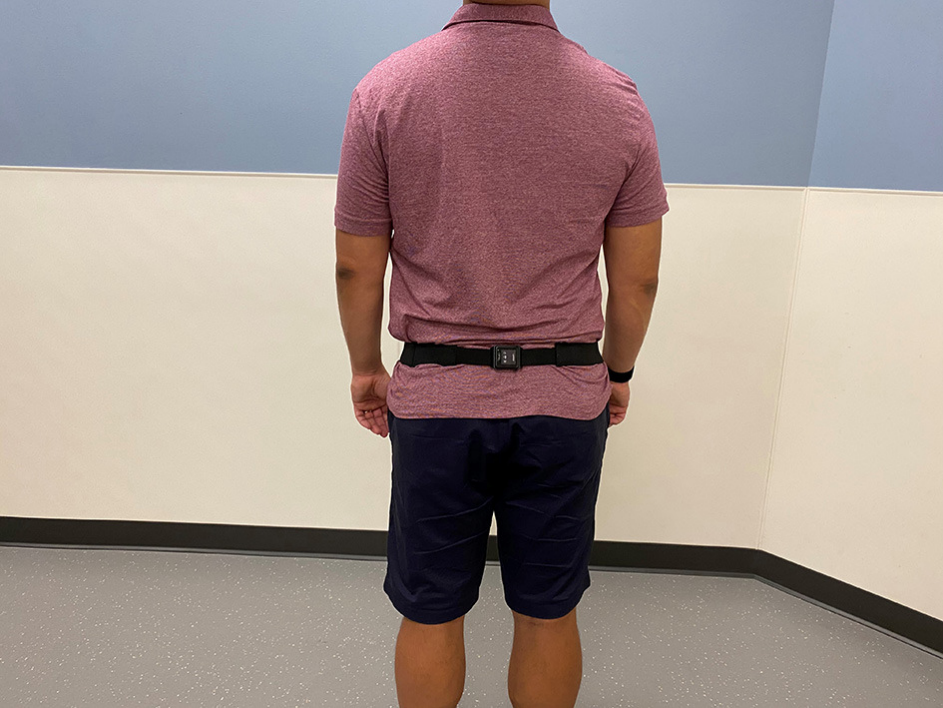
Photograph of waist-worn wearable sensor.

At the baseline visit, the caregiver and the participant were instructed on how to use and charge a commercial wearable sensor, the Opal (APDM, Portland, OR; Figure 1), which was worn on the lower back with an elastic belt against the skin or snuggly around clothing. The Opal is a lightweight (about 22 g) IMU, has a battery life of 12 h, and includes 8 GB of storage. Data from the tri-axial accelerometer and gyroscope was recorded at 128 Hz and stored in the internal memory of the Opal monitors. Participants and/or their caregivers were instructed to wear the device for a minimum of 4 consecutive days for at least 8 h daily during waking hours. The device was battery-operated and charged each night by the participant and/or his/her caregiver. Research staff collected the wearable sensor from the participant's home at the end of the monitoring period, and the devices were cleaned according to manufacturer instructions. The Johns Hopkins Institutional Review Board (IRB) approved this study (Study Number NA_00087648) on 3/18/2014, and informed consent was obtained from the participants and/or their caregivers per established procedures in patients with cognitive impairment (25).

**Figure 2:**
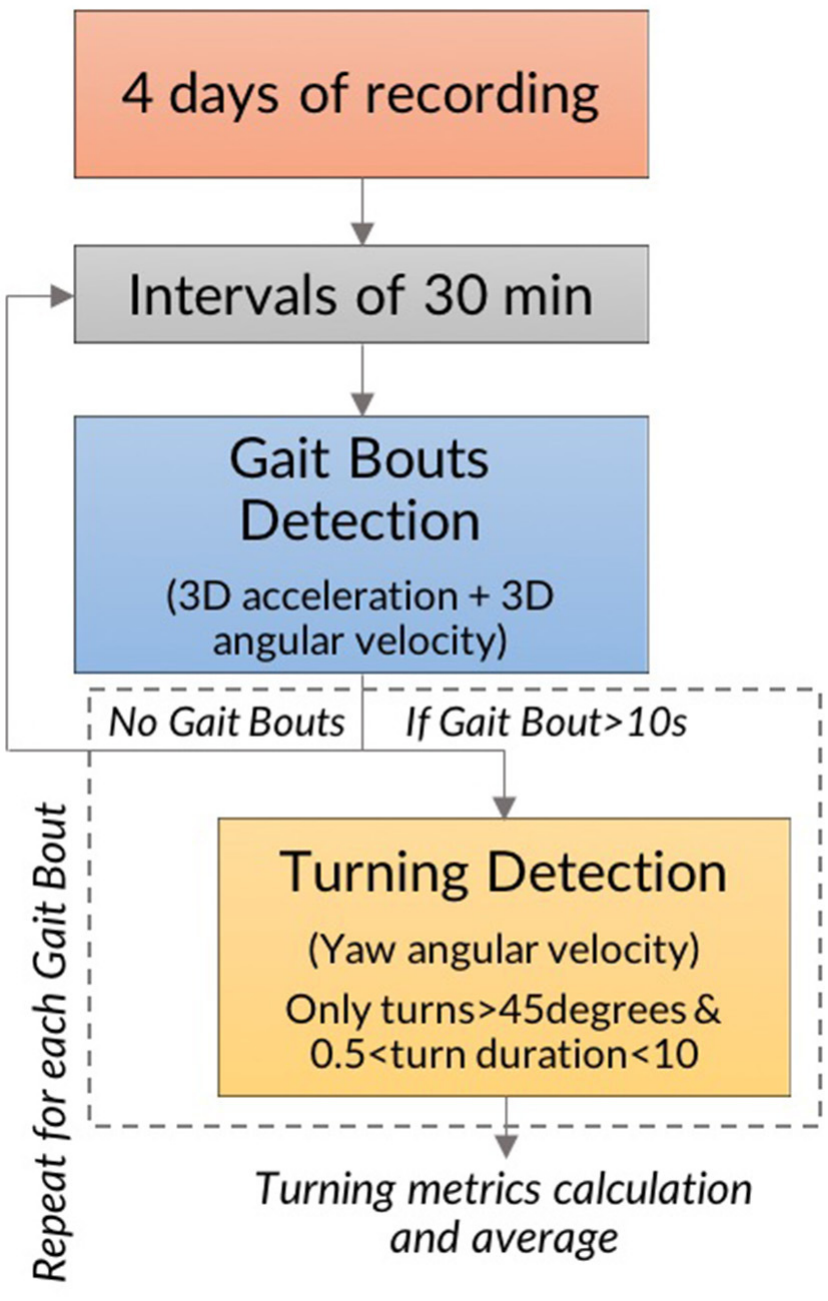
Process for calculation of turning metrics.

Data were downloaded on a laptop and processed in Matlab (R2016b, Mathworks). The process has been previously validated and described (26, 27). A diagram of the algorithm is presented in Figure 2. Gait bouts were defined as periods of walking 10 s or longer as determined by 3D angular velocities and 3D accelerations, in windows of 30 min. Then, the algorithm searched for potential turns within each gait bout by analyzing the horizontal rotational rate. Turning events were defined as a rotation of at least 45 degrees in the horizontal plane (26, 27). Only turning events lasting between 0.5 and 10 s with turn angles of at least 45 degrees were included in the analysis (26, 27). Turn angles were determined by integrating the angular rate of the sensor about the vertical axis (26, 27). The turning characteristics were averaged across time, and data collected included number of hours worn (i.e., total number of analyzed hours, which includes both active and inactive time wearing the sensor), mean number of turns per 30 min interval, mean turn duration, mean peak speed, and mean turn angle (Figure 2).

Participant demographic information was obtained from electronic medical records. The Mini-Mental Status Examination (MMSE) score was obtained at the closest clinic visit to study enrollment. A subset of participants and their caregivers were asked about whether the participants wandered, defined as excessive, repetitive walking without a clear goal or purpose (yes or no). Our data were not normally distributed; therefore, the Spearman rank correlation coefficient was used to determine the strength and direction of the relationship between MMSE score and turning characteristic in our study. Two-sample Wilcoxon rank-sum tests were used to determine if there was a difference in various characteristics between non-wanderers and wanderers in a subset of participants. All analyses were performed using Stata 12.1 (College Station, TX).

## Results

Twelve participants were recruited for this study. The participants were aged 57–85 years [mean 71.5 (±7.26) years], and 5 of 12 (41.7%) were male (Table 1). Participant diagnoses included AD (*n* = 8); vascular dementia (based on a history of multiple strokes, with the diagnosis confirmed by four different neurologists; *n* = 1); Lewy body dementia (DLB) (based on DLB diagnosis criteria laid out by the Consensus Report of the DLB Consortium; *n* = 1) (29); amnestic MCI of AD subtype (based on amyloid positron emission tomography (PET) positivity and diagnosis by a neurologist at the JHMATC; *n* = 1); and dementia due to multiple factors including vasculitis, fibromyalgia, depression, and a previous cerebral vascular accident (diagnosed by a geriatrician at the JHMATC; *n* = 1). MMSE scores ranged from 5 to 29 [mean 18.8 (±7.77)].

**Table 1:** Demographic and turning characteristics.

**Demographic characteristics**					**Turning characteristics**				
**Patient No**.	**Diagnosis**	**Age**	**Gender**	**MMSE^**f**^5 score**	**Number of hours worn**	**Number of turns/30 min**	**Mean turn duration (seconds)**	**Mean peak speed (degree/second)**	**Mean turn angle (degrees)**
1	VD^a^	77	M	21	45.5	5.25	2.53	63.6	83.5
2	AD^b^	79	M	29	16.5	4.73	1.86	69.3	68.2
3	AD	85	M	14	33.5	1.82	1.56	112.8	82.3
4	LBD^c^	69	M	27	28.5	1.20	2.45	61.2	68.3
5	MCI^d^	67	F	25	42.0	26.8	2.24	80.8	102.5
6	AD	69	F	20	21.0	6.70	2.02	103.7	97.5
7	Multifactorial^e^	71	F	25^*^	34.0	122.0	1.70	99.4	92.8
8	AD	76	F	23^*^	35.5	95.0	2.32	64.7	89.0
9	AD	65	F	8	42.5	64.0	1.79	83.0	80.6
10	AD	70	F	5	26.0	88.0	2.04	66.7	81.6
11	AD	73	F	18	30.5	48.0	2.27	78.3	96.7
12	AD	57	M	11^*^	30.5	76.0	2.05	80.2	93.1
Overall, mean (SD)					32.2 (8.66)	45.0 (43.1)	2.07 (0.30)	80.3 (17.0)	86.3 (11.0)
**Data from Mancini et al. study** **(****23****)**									
Non-fallers, mean (SD)					–	31.8 (8.95)	2.11 (0.17)	75.9 (4.14)	95.2 (2.41)
Recurrent fallers, mean (SD)					–	23.1 (7.10)	2.42 (0.26)	65.6 (9.50)	92.5 (7.21)
Spearman rank correlation coefficient between MMSE score and turning characteristic, *r* (*p*-value)					−0.11 (0.74)	−0.33 (0.30)	0.23 (0.47)	−0.28 (0.38)	−0.08 (0.80)

a *VD, vascular dementia*.

b *AD, Alzheimer's disease*.

c *LBD, Lewy body dementia*.

d *MCI, mild cognitive impairment*.

e *Multifactorial, Dementia thought to be due to depression, vasculitis, fibromyalgia, and past Cerebrovascular accident (CVA)*.

f *MMSE, Mini-Mental Status Examination*.

* *Montreal Cognitive Assessment (MoCA) scores were obtained in the clinic, and equivalent MMSE scores were reported in this chart (28)*.

*Participants were dementia patients seen at the Memory Clinic at the Johns Hopkins Department of Geriatrics in 2016–2017*.

All 12 participants wore the device as instructed. Participants wore the device a mean of 32.2 (±8.66) h over the course of 4 days (an average of 8 h daily). The average data from Mancini et al.'s study are described in Table 1 for direct comparison to our findings (23). Comparing our data to Mancini et al.'s study of older adults with and without cognitive impairment, our cohort of participants with cognitive impairment trended toward having a greater number of turns in 30 min, a shorter mean turn duration, a faster mean peak turning speed, and a smaller mean turn angle (Table 1). We also evaluated the Spearman correlation between the MMSE score of our participants and each turning characteristic. We did not observe any significant correlations between MMSE score and length of device use or turning characteristics in this small sample.

**Figure 3:**
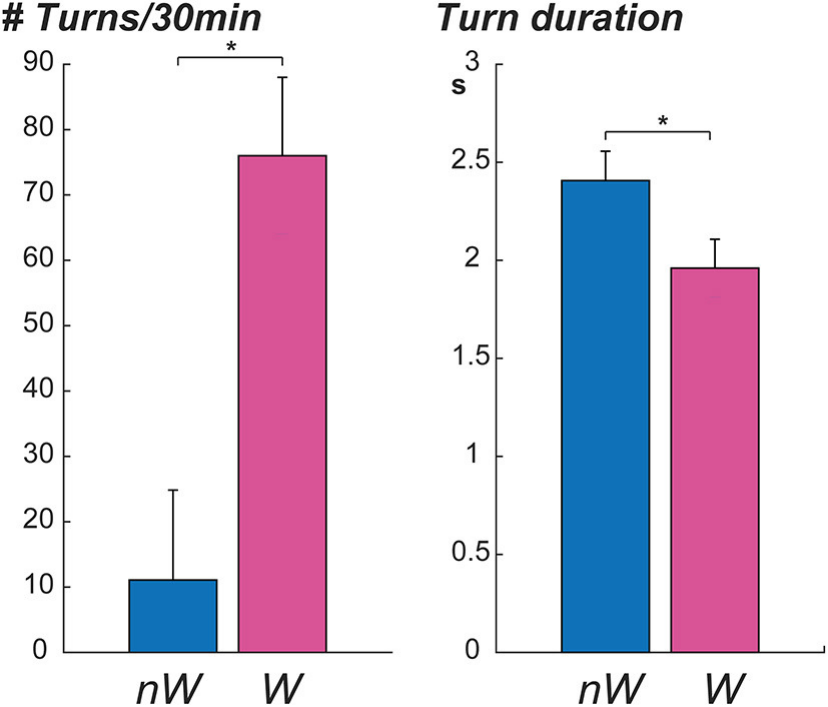
Comparison of turning metrics between wanderers (W) and non-wanderers (nW).

Six participants and caregivers provided information about wandering behaviors; three participants were reported to wander by their caregivers, and three participants were reported not to wander. Participants who wandered had significantly lower MMSE scores, higher number of turns in 30 min, and shorter mean turn duration (Table 2). No significant associations were observed between wandering and age, mean peak speed, or mean turn angle. Graphs displaying the mean number of turns in 30 min and mean turn duration of non-wanderers (NW) and wanderers (W) are seen in Figure 3. Asterisks denote a significant *p*-value.

**Table 2:** Demographic and turning characteristics in non-wanderers vs. wanderers.

**Demographic and turning characteristics**	**Non-wanderers (*n* = 3)**	**Wanderers (*n* = 3)**	**Z-score**	***p*-value^**a**^**	**Probability non-wanderers > wanderers^**b**^**
	**Mean (SD)**	**Mean (SD)**			
Age (years)	71.0 (5.29)	64.0 (6.56)	1.09	0.275	77.8%
MMSE score	24.3 (3.06)	8.00 (3.00)	1.96	0.0495	100%
Number of turns/30 min	11.1 (13.8)	76.0 (12.0)	−1.96	0.0495	0.00%
Mean turn duration (seconds)	2.41 (0.15)	1.96 (0.15)	1.96	0.0495	100%
Mean peak speed (degree/second)	68.5 (10.7)	76.6 (8.72)	−1.09	0.275	22.2%
Mean turn angle (degrees)	84.8 (17.2)	85.1 (6.95)	0.218	0.827	55.6%

a *Two-sample Wilcoxon rank-sum tests were used to determine if there was a difference between non-wanderers and wanderers. A p-value < 0.05 is considered significant*.

b *The probability that the value of the demographic and turning characteristic of non-wanderers is greater than wanderers*.

## Discussion

In this feasibility study, people with MCI and dementia tolerated continuous monitoring of mobility with a wearable sensor and provided evaluable data on turning behavior. Moreover, in a small subset of participants, we observed that participants who wandered had a significantly shorter mean turn duration and higher turn rate (number of turns/30 min) over the entire time they were wearing the device relative to participants who did not wander. Although our findings are preliminary, this feasibility study showed that detailed information about the quality of motor behavior under real-life conditions can be collected, such as mean peak speed, mean turn duration, and mean turn angle, in patients with cognitive impairment. In this small sample, we did not observe that severity of cognitive impairment was associated with duration of device use or with any turning characteristics.

Several prior studies have tracked abnormal motor behavior including wandering in patients with dementia. Wandering behavior includes lapping, pacing, directionless movements, and frequently getting lost. One study used wearable sensors to track gait and balance in the laboratory in AD patients (15). Other studies measured path tortuosity using a fractal dimension detected by a sensor network in an assisted living facility occupied by older adults (30, 31). Another group developed an algorithm to detect lapping and pacing wandering behavior using mobile health technology, although this has yet to be validated in patients (5, 32). Lin et al. used GPS traces from GPS-equipped cell phones to define pacing and lapping movements by summing the angles of turning points in a given trajectory and using this value to decide if the movement qualifies as pacing or lapping. However, to the best of our knowledge, no previous study characterized turning while walking in people with cognitive impairment in the home environment over multiple days. This small study demonstrates the feasibility of continuous monitoring in patients with cognitive impairment and the potential of using one IMU to objectively define and quantify wandering behavior.

Our data suggest that individuals who wander appear to make more frequent, shorter turns relative to individuals who do not wander, providing further insight into the wandering behavioral phenotype. These findings are consistent with the observation that wanderers often perform pacing and lapping behavior, which could lead to more frequent turns. Wanderers may make shorter turns due to more frequent directionless movement when compared to non-wanderers. Moreover, wanderers often get lost, which could be reflected in more frequent, faster turns employed to find their way or attempt to reorient themselves. However, our sample size is very small, and the extent to which these differences may reflect differences in total motor activity is unclear. Future studies in larger samples will be needed to more definitively establish the relationship between wandering and turning characteristics with more objective measures of wandering such as video surveillance.

Limitations of this study were the small sample size and the inclusion of participants with various etiologies of cognitive impairment. Additionally, although the same device and analysis were used for the control group, the control group was part of a separate study by one of the investigators, which may have limited the comparability of the groups. In future work, we plan to evaluate whether objective measures of turning behaviors collected using the inertial sensor can be used to identify wandering and other abnormal motor behaviors in patients with dementia. Moreover, in a larger sample size, we will consider other characteristics of motor behaviors such as diurnal variability.

## Data Availability Statement

The datasets generated for this study are available on request to the corresponding author.

## Ethics Statement

The studies involving human participants were reviewed and approved by Johns Hopkins Institutional Review Board. The patients/participants provided their written informed consent to participate in this study.

## Author Contributions

YA, MM, DB, GX, and RK contributed to study concept and design, data analysis, and manuscript preparation. EO, EW, and AP participated in data collection. All authors contributed to the article and approved the submitted version.

## Conflict of Interest

The authors declare that the research was conducted in the absence of any commercial or financial relationships that could be construed as a potential conflict of interest.
